# Design and methodology of the screening for CKD among older patients across Europe (SCOPE) study: a multicenter cohort observational study

**DOI:** 10.1186/s12882-018-1030-2

**Published:** 2018-10-11

**Authors:** Andrea Corsonello, Lisanne Tap, Regina Roller-Wirnsberger, Gerhard Wirnsberger, Carmine Zoccali, Tomasz Kostka, Agnieszka Guligowska, Francesco Mattace-Raso, Pedro Gil, Lara Guardado Fuentes, Itshak Meltzer, Ilan Yehoshua, Francesc Formiga-Perez, Rafael Moreno-González, Christian Weingart, Ellen Freiberger, Johan Ärnlöv, Axel C. Carlsson, Silvia Bustacchini, Fabrizia Lattanzio, on behalf of SCOPE investigators, Fabrizia Lattanzio, Andrea Corsonello, Silvia Bustacchini, Silvia Bolognini, Paola D’Ascoli, Raffaella Moresi, Giuseppina Di Stefano, Laura Cassetta, Anna Rita Bonfigli, Roberta Galeazzi, Federica Lenci, Stefano Della Bella, Enrico Bordoni, Mauro Provinciali, Robertina Giacconi, Cinzia Giuli, Demetrio Postacchini, Sabrina Garasto, Annalisa Cozza Romano Firmani, Moreno Nacciariti, Mirko Di Rosa, Paolo Fabbietti Roberto Bernabei, Christophe Bula, Hermann Haller, Carmine Zoccali, Kitty Jager, Wim Van Biesen, Paul E. Stevens

**Affiliations:** 1 Italian National Research Center on Aging (INRCA), Ancona, Fermo and Cosenza Italy; 2 Section of Geriatric Medicine, Department of Internal Medicine, Erasmus University Medical Center Rotterdam, Rotterdam, The Netherlands; 3 Department of Internal Medicine, Medical University of Graz, Auenbruggerplatz 15, 8036, Graz, Austria; 4 CNR-IFC, Clinical Epidemiology and Pathophysiology of Hypertension and Renal Diseases, Ospedali Riuniti, Reggio Calabria, Italy; 5 Department of Geriatrics, Healthy Ageing Research Centre, Medical University of Lodz, Lodz, Poland; 6 Department of Geriatric Medicine, Hospital Clinico San Carlos, Madrid, Spain; 7 The Recanati School for Community Health Professions at the faculty of Health Sciences, Ben-Gurion University of the Negev, Beersheba, Israel; 8 Maccabi Healthcare Services Southern Region, Tel Aviv, Israel; 9 Geriatric Unit, Internal Medicine Department and Nephrology Department, Bellvitge University Hospital – IDIBELL – L’Hospitalet de Llobregat, Barcelona, Spain; 10 Department of General Internal Medicine and Geriatrics, Krankenhaus Barmherzige Brüder Regensburg and Institute for Biomedicine of Aging, Friedrich-Alexander-Universität Erlangen-Nürnberg, Erlangen, Germany; 11 Department of Medical Sciences, Uppsala University, Uppsala, Sweden; 12 School of Health and Social Studies, Dalarna University, Falun, Sweden; 13 Division of Family Medicine, Department of Neurobiology, Care Sciences and Society, Karolinska Institutet, Stockholm, Sweden

**Keywords:** Chronic kidney disease, Older people, Disability, Frailty, Ageing

## Abstract

**Background:**

Decline of renal function is common in older persons and the prevalence of chronic kidney disease (CKD) is rising with ageing. CKD affects different outcomes relevant to older persons, additionally to morbidity and mortality which makes CKD a relevant health burden in this population. Still, accurate laboratory measurement of kidney function is under debate, since current creatinine-based equations have a certain degree of inaccuracy when used in the older population. The aims of the study are as follows: to assess kidney function in a cohort of 75+ older persons using existing methodologies for CKD screening; to investigate existing and innovative biomarkers of CKD in this cohort, and to align laboratory and biomarker results with medical and functional data obtained from this cohort. The study was registered at ClinicalTrials.gov, identifier NCT02691546, February 25th 2016.

**Methods/design:**

An observational, multinational, multicenter, prospective cohort study in community dwelling persons aged 75 years and over, visiting the outpatient clinics of participating institutions. The study will enroll 2450 participants and is carried out in Austria, Germany, Israel, Italy, the Netherlands, Poland and Spain. Participants will undergo clinical and laboratory evaluations at baseline and after 12 and 24 months- follow-up. Clinical evaluation also includes a comprehensive geriatric assessment (CGA). Local laboratory will be used for ‘basic’ parameters (including serum creatinine and albumin-to-creatinine ratio), whereas biomarker assessment will be conducted centrally. An intermediate telephone follow-up will be carried out at 6 and 18 months.

**Discussion:**

Combining the use of CGA and the investigation of novel and existing independent biomarkers within the SCOPE study will help to provide evidence in the development of European guidelines and recommendations in the screening and management of CKD in older people.

**Trial registration:**

This study was registered prospectively on the 25th February 2016 at clinicaltrials.gov (NCT02691546).

## Background

Evidence from epidemiological and clinical literature suggests that ageing contributes to the incidence of reduced renal filtration capacity [1]. In the presence of risk factors during ageing, such as diabetes, hypertension and others, filtration capacity further declines. This concept is underlined by many epidemiological studies showing a decline of measured estimated glomerular filtration rate (eGFR) with advancing age [2]. Kidney function is usually assessed by creatinine-based estimated glomerular filtration rate (eGFR) equations. However, those formulae have a certain degree of inaccuracy when used in older people due to changes in anthropometry and renal physiology during ageing [3]. Alternative filtration markers yielded different eGFR values for different cohorts of people tested [4]. This inaccuracy of laboratory measurements of kidney function suggests a risk of underdetection or overdetection of CKD, especially with advancing age [5]. Indeed, the eGFR threshold at which the risk of negative outcomes increases among older patients is hotly debated [6], and current evidence suggests that such a threshold may be lower among older people compared to adult ones [7–9]. Additionally, the eGFR cut-offs at which the risk of death starts to increase may change as a function of the equation used among older people [10]. Thus, improving accuracy of CKD screening measures for older populations would be of help in reducing the risk of underdiagnosis to maximize prevention of CKD and its consequences while minimising the risks and cost of overdiagnosis [6].

Diminished kidney function has become a relevant public health burden for all age groups, as CKD frequently results in an increased risk of end stage renal disease (ESRD), morbidity and mortality [11]. Besides “traditional” endpoints, CKD has been shown to impact nutritional status, inflammatory processes and anemia [12], thereby affecting different outcomes especially relevant to older people. These include impaired physical function, frailty and disability [13–16], cognitive impairment and dementia [17–19], depression [20–22], sensory impairment [23], undernutrition and sarcopenia [24–26], and adverse drug reactions (ADRs) [27, 28]. Therefore, early and sensitive detection of diminished renal function is essential to individually address care needs of older people with CKD and to address one of the major health burden in public health for the incoming decades [29].

Incorporating scoring risk models for care planning of older people at risk for CKD has come into focus recently [30]. Risk prediction models are generally based on equations designed on the basis of prognostic factors and clinical outcomes, available at the time the prediction is made, and collected in specific and representative cohorts of individuals followed up for a given period of time [31]. Built on evidence of such models, screening programmes for CKD can take into account the characteristics of the target population in addition to simple laboratory measures, biomarkers and disease-based investigations. Multi- and co-morbidity, polypharmacy, frailty, functional and cognitive impairment and disability should be considered as part of a patient centered approach in CKD management especially in older adults [15, 23–26, 32–35].

So far, no CKD screening program has included all those variables also including data from comprehensive geriatric assessment (CGA), the only assessment technology able to capture the numerous domains of health status and their complex interactions in older people. Accordingly, the need for laboratory measurements able to identify accurately older people with CKD is a demand to address the public health challenges arising from the current demographic shifts. Indeed, this view is widely shared by the geriatric and nephrology communities, both in EU and USA [36, 37].

The aims of this multicenter study in Europe are to assess existing methodologies for CKD screening and investigate existing and innovative biomarkers of CKD in older persons. Furthermore, the Screening for CKD among Older People across Europe (SCOPE) study will provide evidence for including physical and functional health parameters of older people across Europe and help design a tailored risk prediction model for CKD in old age.

## Methods

### Study design

**Fig. 1:**
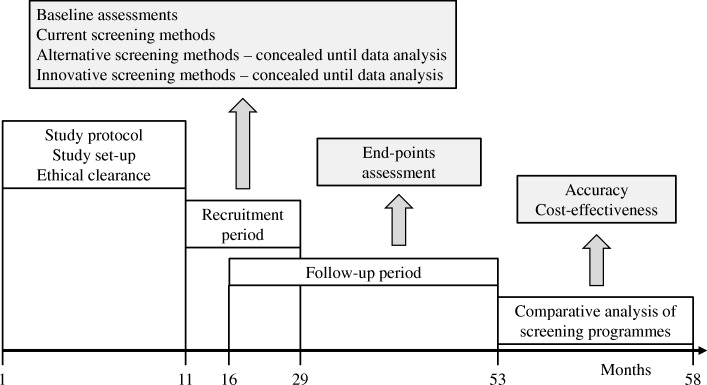
Study design of the SCOPE project

The SCOPE study is designed as an observational, multinational, multicenter, prospective cohort study in persons older than 75 years across Europe. This study is carried out in seven countries, including Austria, Germany, Israel, Italy, the Netherlands, Poland and Spain. Participants will undergo clinical and laboratory evaluations at the baseline (recruitment), and will be followed up at face to face visits at months 12 and 24 following enrollment. An intermediate telephone follow-up will be carried out at 6 and 18 months following recruitment. Figure 1 shows the schematic flow of the observational clinical study.

The study design complies with the Declaration of Helsinki and Good Clinical Practice Guidelines. The enrollment has started in August 2016 and is ongoing.

### Ethical approval/ monitoring

The study protocol was approved by ethics committees at all participating institutions. Patients are requested to sign a written informed consent before entering the study. Patients are also asked to sign a separate informed consent to the collection of DNA samples to be used for genetic testing, while those not giving their consent will be retained in the main cohort study.

In order to ensure high ethical and scientific standards of the project and to monitor the progress of the clinical study a Scientific Advisory Board (SAB) and a Data and Ethics Management Board (DEMB) was implemented within the Governance Structure. The SAB ensures a high standard of research, monitors the progress of the project by taking part in the project meetings, and provides final approval to any required study amendments. The DEMB supports the preparation of the relevant end-points for ethical review, advises on local research Ethical Committee applications, and reviews the relevant safety, morbidity and mortality end-points during the course of the study. The DEMB maintains an overview of the work throughout the whole course of the project and helps to foresee possible problems that might arise and how they can be addressed.

### Study population

Persons aged 75 years and older, visiting the outpatient clinics of participating institutions are eligible for inclusion. The study design aims at minimizing self-selection bias and enrolling real-world patients without stringent inclusion/exclusion criteria. The few exclusion criteria are outlined in Table 1. Therefore, no other inclusion criteria will be considered. The SCOPE study aims to finally enroll 2450 participants.

**Table 1:** Exclusion criteria for participants enrollment into the SCOPE project

• Age < 75 years	
• End stage renal disease (< 15 mL/min/1.73 m2) or dialysis at time of enrollment	
• History of solid organ or bone marrow transplantation	
• Active malignancy within 24 months prior to screening or metastatic cancer	
• Life expectancy less than 6 months	
• Severe cognitive impairment (Mini Mental State Examination < 10)	
• Any medical or other reason (e.g. known or suspected inability of the patient to comply with the protocol procedure) in the judgement of the investigators, that the patient is unsuitable for the study	
• Unwilling to provide consent and those who cannot be followed-up	

### Study visits

Following enrollment, participants will be seen by the study teams at 12 and 24 months at a face to face meeting. Demographic data and socioeconomic status (occupation before retiring, economic status, formal and informal care) will be documented and followed up at each visit. Physical examination will be performed by medical doctors due to standardized procedure given in the visit protocol. Medical history and use of medication and adverse drug reactions classified according to the World Health Organization (WHO) definition [38] will be collected during follow-up visits. During all face to face visits a comprehensive geriatric assessment (CGA) will be performed. Table 2 shows all domains checked during study visits [39–51] [52].

**Table 2:** Comprehensive Geriatric Assessment domains tested during the SCOPE project

• Basic (ADL) and Instrumental Activities of Daily Living (IADL)/self-reported disability [39, 40]	
• Mini Mental State Examination (MMSE)/cognitive status [41]	
• 15-items Geriatric Depression Scale (GDS)/mood [42]	
• Cumulative Illness Rating Scale (CIRS)/overall comorbidity [43]	
• History of falls and incident falls	
• Vision and hearing impairment will be coded on a scale from 0 (adequate) to 4 (no vision/hearing present) [44].	
• Lower urinary tract symptoms (LUTS): The presence of LUTS will be ascertained by asking the patient to rate on a 5-point (0–4) Likert scale how big a problem, if any, has each of the following items been during the last 4 weeks: 1. Dripping or leaking urine, 2. Pain or burning in urination, 3. Bleeding with urination, 4. Weak urine stream or incomplete emptying, 5. Waking up to urinate, 6. Need to urinate frequently during the day [45].	
• Nutritional status: anthropometric parameters (calf circumference, arm circumference, Body mass index (kg/m2), waist-hip ratio, waist-to-height ratio), Mini Nutritional Assessment (MNA) [46] and 24-h dietary recall^a^ [47].	
• Short Physical Performance Battery (SPPB) [48].	
• Grip strength [49] measured by using JAMAR hydraulic dynamometer.	
• Bioelectrical impedance analysis (BIA)^b^ [50] Muscle mass will be calculated using the Janssen et al. equation [51], using the instrument Akern BIA101.	
• Health related quality of life will be rated by the Euro-QoL 5D.	

^a^Data obtained from the 24-h dietary recall will be analyzed using nutritional databases suitable for the patient’s country. Following the analysis, a detailed report (containing levels of consumption of various nutrients and energy) will be available. This level will be compared with recommended levels of intake

^b^BIA will not be performed in patients with pacemaker or implantable cardioverter defibrillator

Healthcare resource consumption will be evaluated using a resource use questionnaire within a 6-month recall time-frame [50]. Following information will be retrieved: previous physician visits (GPs, specialists, or physician at the Emergency Room), use of diagnostic tests and specialist clinic procedures, use of care services (e.g. Nurse home visit, Physiotherapy, Home help, Social transport, Day care center) and hospital admissions (number and duration of hospitalization, type of reimbursement).

Furthermore, caregiver burden will be measured using the Zarit Burden Interview (ZBI) [53].

During enrollment and at the two face to face follow up visits blood and urine samples will be collected and analysed for serum creatinine, urinary albumin and albumin-to-creatinine ratio.

### Telephone follow-up

At 6- and 18-month participants and/or caregivers will be interviewed by phone to collect information on vital and functional status and healthcare resource consumption. Changes in medical history and adverse drug reactions will also be collected.

### Laboratory parameters and biomarkers

Serum creatinine measurement will be standardised to Isotope-Dilution Mass Spectrometry at local level, when the method is available. Creatinine-based eGFR will be calculated using the Berlin Initiative Study 1 (BIS1) equation, which is the only method specifically developed in a population older than 70 years [54]. ESRD will be defined as GFR < 15 mL/min/1.73 m2 or dialysis [55]. In case of unavailability of standardized creatinine methodology at local level, this measurement will be made by INRCA laboratories afterwards. The panel of laboratory parameters to be measured at baseline, 12-month and 24 months by local laboratories will also include: complete blood cells count, lipids profile, electrolytes, nutritional status, and urine analysis.

The project will also include the collection of blood and urine samples to investigate existing and innovative biomarkers of kidney function. Existing biomarkers of CKD like Cystatin C (CysC) [56], β-Trace protein (BTP), also known as lipocalin prostaglandin D2 synthase [57, 58] Beta2-microglobulin [59] will be measured using published and established methods. Potential and new biomarkers will be also evaluated. Furthermore, the evaluation of experimental kidney damage biomarkers as well as untargeted analysis of metabolomics in serum and urine is currently be performed in ULSAM [60] and PIVUS [61] studies, in order to identify additional kidney damage biomarkers that may be validated in the SCOPE project. Table 3 shows an overview on current, alternative and innovative biomarkers for CKD whose applicability in old age will be investigated within the SCOPE project.

**Table 3:** Biomarkers research in the SCOPE project

Current screening methods^a^	Alternative screening methods^b^	Innovative screening methods^b^
Serum creatinine	Serum cystatin C	Serum fibroblast growth factor 23
Creatinine-based eGFR	Serum β-trace protein	Serum and urinary soluble TNF receptor 1
Urinary albumin	Serum β2-microglobulin	Seerum and urinary soluble TNF receptor 2
Albumin-to-creatinine ratio		Serum and urinary osteopontin
		Serum penthraxin 3
		Serum and urinary endostatin
		Serum and urinary TIM-1 (KIM-1)
		Serum TRAIL R2
		Serum and urinary endostatin

^a^current screening measures will be assessed at local laboratories and are immediately available after enrollment and follow-up visits;

^b^alternative and innovative screening measures will be centrally assessed and will be concealed until data analysis

The assessment of selected genetic and epigenetic parameters involved in hallmarks of aging will be also carried out to investigate their relationship with kidney function. This latter assessment will be limited to participants who signed a separate informed consent (patients not giving informed consent for genetic and epigenetic analysis will be retained in the main cohort study), and will include: DNA methylation, polymorphisms of mitochondrial DNA, polymorphisms of genes coding for pro- and anti-inflammatory cytokines (IL-6, IL-1, TNF-alpha, IL-10, IL-2, IL-17, IL-8) and chemokines (MCP-1 and RANTES), polymorphisms associated with molecules involved in the pathogenesis of metabolic and neurodegenerative diseases such as insulin and IGF-1 signaling pathway and APOE, Klotho, mTOR, and whole genome analysis by Affymetrix Chip Array 6.0.

### Measured glomerular filtration rate

The assessment of measured glomerular filtration rate (mGFR) will be performed by single-dose inulin clearance [62, 63]. Participants will be asked to sign a separate informed consent to participate in this sub-study, while those not giving their consent will be retained in the main cohort study. The objective of this sub-study will be the derivation of new eGFR equation(s) based on already known and/or novel biomarkers. The accuracy of new equation(s) in predicting mGFR will represent the primary study endpoint. Accuracy will be assessed by P30 (percentage of estimates within 30% of the mGFR). A sample of 400 participants will enable us to detect a difference of 2% in P30 between the new equations (based on the innovative and novel biomarkers) and the BIS equations, with significance level 0.05 and power 0.8 (considering a 1-sample and 1-sided test). In addition, we have evaluated that the sample will be sufficient to detect a statistically significant difference in 4,3 points in the Area under the ROC curve using the new equation(s) for discriminating participants below the critical threshold of 60 ml/min/1.73 m^2^. Finally, the availability of mGFR in a subgroup of participants enrolled in the study will be used to investigate the relationship between innovative biomarkers and objectively measured kidney function.

### Study endpoints

The primary study endpoints will be the rate of eGFR decline and the incidence of ESRD.

The secondary endpoints will include measures of conventional and geriatric outcome measures, such as: rate of CKD complications (anemia, hyperphosphatemia, acidosis, hypoalbuminemia, hyperparathyroidism, hyperkaliemia); rate of major comorbidities (e.g. hypertension and CV diseases) [43]; overall and CV mortality; adverse drug reactions (ADRs); self-reported disability and objectively measured physical performance decline [39, 40, 48]; cognitive impairment [41]; depression [42]; malnutrition/undernutrition [46, 47]; health-related quality of life [52]; healthcare resource consumption, including the estimation of caregiver burden [53].

Informations on vital status during follow-up will be obtained by interviewing the patients and/or their formal and/or informal caregivers. For mortality during the follow-up period, date, place and cause of death will be retrieved by certificates of death exhibited by relatives or caregivers.

### Data management and statistics

The SCOPE project will enroll a total of 2450 participants. On the basis of the primary end-points, a sample of 1900 patients will be able to differentiate between two equally sized subgroups according to a standardized difference in yearly rate of GFR decline of 0.13 mL/min/1.73m^2^ with a power of 80%. The same sample size allows to detect a hazard ratio of 1.2 in time-to-event analyses with 80% power for incidence of ESRD. Thus, even a 20% drop out rate will not affect statistical power of the study.

Every effort will be made to collect all data at the specified time points. In the case of missing (and not recoverable) data on primary endpoints, we will make the assumption that data are missing completely at random. Analyses will be carried out applying the list-wise deletion of cases with missing values in order to obtain unbiased estimations. Multiple imputation of missing data will be applied only for secondary endpoints and co-variates when found appropriate.

For continuous outcomes, generalized mixed models will be used while for dichotomous outcomes, random effect logistic or Cox regression will be applied. Effect modification by age and gender will be investigated using multiplicative interaction analyses.

Relevant exposure and co-variates will be selected based on plausible underlying hypothesis. Directed acyclic graphs may be used in order to create parsimonious multivariable models with minimized confounding. If appropriate, repeated measurements of exposure and co-variates will be included in the models.

### Economic monitoring

The economic analysis of the SCOPE project will include: i) cost of screening/diagnosis; ii) cost of follow-up (e.g. pharmacological treatment, specialist visits, laboratory visits over the 2-year follow-up); iii) cost of CKD complications (e.g. emergency room access, hospital admission, haemodialysis, etc.); iv) other health-related costs (e.g. hospital out-patient care referrals, nursing home placements, use of home care services). With this analysis, it will be possible to determine main predictors of costs in CKD using multivariate regression and to establish cost-effective ratio of the intervention (overall healthcare costs, divided by efficacy, expressed as survival or quality-adjusted survival).

In order to assess the cost-benefit profile of the screening program on a longer time horizon, clinical and economic results of the SCOPE project will be used to run a projection (10–15 years) using Markov modelling. The analysis consists in evaluating a hypothetical cohort of CKD patients, whose healthcare status is categorized into different initial Markov states, based on CKD biomarkers. Patients can move from one state to another, according to certain probabilities that will be derived from the SCOPE project, and can develop complications, such as cardiovascular morbidity, renal failure and need of dialysis, CKD related and non-related death.

## Discussion

The SCOPE study is one of the largest prospective observational cohort studies aimed at screening for CKD among older persons across Europe. The current paper outlines the study protocol including statistical analysis of data, risk prediction modeling and economic evaluation of costs arising from CKD during the advanced ageing process.

The strength of the protocol outlined in this paper is the real life setting for recruitment of participants. All persons with age ≥ 75 years attending the outpatient services at participating institutions will be requested to participate in the study. No other inclusion criteria will be considered. This seems the primary strength of the SCOPE study. The collection of real life data in a longitudinal fashion over a two- years period of time will allow insight on the impact of renal function on the management and advanced care planning of older subjects prone to renal impairment.

It is expected that many of the participants enrolled will be affected by multimorbidity [64]. The impact of disease clusters and management strategies from experts in the field of nephrology and geriatrics will open access to comparative effectiveness analysis of data and interventions [65]. People older than 75 years or people with impaired renal function have so far been rarely included into clinical trials. Aging population heralds a new geriatric “reality”, namely an increase in older adults with CKD. Conversely, many older adults are living healthy and active, even with several chronic conditions. In this context longitudinal epidemiological studies are extremely valuable tools in observational research and have many uses and strengths [66].

Multimorbidity, and in this context CKD have been shown to impact functional status, especially of older patients [66]. The systematic use of a CGA makes possible the investigation of multiple domains of health status in older persons. CGA is part of clinical practice of Geriatric Medicine [67] and is also useful in research investigating consequences of CKD [68, 69] since it has been shown to affect different kind of outcomes relevant to older people. The inclusion of functional domains, as recently postulated by the World Health Organization (WHO) [70] in the design of screening models for CKD in older persons aligns the SCOPE projects with future demands for all Health Care systems around the globe [71, 72]. Health care is currently provided and funded on a disease-centered approach in many health care systems. The inclusion of CGA in the longitudinal evaluation of study participants of the SCOPE project will allow a more patient-centered and individualized approach for screening and advanced care planning for older subjects prone to kidney function decline [31, 69]. Furthermore, the search for biomarkers which are less influenced by muscle mass and more accurate in predicting outcomes compared to circulating creatinine is of special interest and will be further investigated. Thus, combining the use of CGA and the investigation of novel and existing independent biomarkers in within the SCOPE project, could help in building new evidence in the development of recommendations and guidelines for a patient-centered approach in the screening and management of older people at risk for CKD.

The alignment of an economic evaluation of care pathways and histories of study participants during the study period will give new input for care providers and planners in different health care and funding systems. Inclusion of costs of screening to achieve accurate diagnosis of CKD and related follow-up costs (e.g. pharmacological treatment, specialist visits, laboratory visits over the 2-year follow-up) will answer current call for actions coming from different bodies [73]. The focus on CKD related consumption of healthcare resources (e.g. emergency room access, hospital admission, hospital out-patient care referrals, nursing home placements, use of home care services and others) using Markov modelling will provide key information for developments in public health.

Major drawback or limitation of the project is the lack of standardized management and care plans for older people currently available for all participating centres. Centres enrolling participants in the SCOPE projects are highly experienced in the management of older multimorbidity subjects at risk for renal impairment and related clinical complications, including changes in functional status. Guidelines on CKD management are mainly disease-centred and put a focus on morbidities and mortality. It is to be foreseen that the care pathways for participants will therefore still be tailored individually and according to needs, driven by expertise of staff in the participating centres. However, important information may be expected though, as the implementation of the CGA per se into care pathways has already been proven effective [67]. It seems noteworthy that the individualized care approach during complex care management of older subjects is part of daily routine in geriatric medicine. Alignment of care processes along CGA results seems feasible in the context of current scientific evidence.

In conclusion, the SCOPE project will close essential gaps in the care of older people with declining kidney function. Due to the extremely comprehensive study setting and data analysis it is to be expected that evidence arising from the SCOPE project will impact the management of older people suffering from CKD, as well as the quality of care delivered for older subjects at risk for CKD in daily routine. The high quality of data retrieved will however, also open doors for new research and innovation in the field of nephrology and geriatrics. Building on solid evidence arising from the current project, SCOPE will support the development of European recommendations and guidelines, as well as a European education program in the field of screening and management of CKD in older adults across Europe.

## Abbreviations

ADL: Activities of Daily Living
ADRs: Adverse drug reactions
APOE: Apolipoprotein E
BIA: Bio-impedance analysis
BIS: Berlin Initiative Study
BTP: Beta-trace proetin
CGA: Comprehensive geriatric assessment
CKD: Chronic kidney disease
CysC: Cystatin C
DEMB: Data and Ethics Management Board
eGFR: Estimated glomerular filtration rate
ESRD: End-stage renal disease
GDS: Geriatric Depression Scale
IADL: Instrumental Activities of Daily Living
IL: Interleukin
LUTS: lower urinary tract symproms
MCP-1: Monocyte Chemoattractant Protein-1
MMSE: Mini Mental State Exam
MNA: Mini Nutritional Assessment
mTOR: Mammalian target of rapamycin
PIVUS: Prospective Investigation of Vasculature in Uppsala Seniors
RANTES: Regulated on Activation, Normal T Cell Expressed and Secreted
SAB: Scientific Advisory Board
SCOPE: Screening for CKD among Older People across Europe
SPPB: Short Physical Performance Battery
TNF: Tumor Necrosis Factor
ULSAM: Uppsala Longitudinal Study of Adult Men
WHO: World Health Organization
ZBI: Zarit Burden Interview
